# Role of prior HPV infection and CD4 T-cell count in modulating cellular immune responses to a three-dose nonavalent HPV vaccine schedule in PWH receiving ART

**DOI:** 10.1186/s12916-025-04504-1

**Published:** 2025-12-24

**Authors:** Eeva Tortellini, Mariasilvia Guardiani, Anna Carraro, Lorenzo Ansaldo, Sara Corazza, Sara Giovanna De Maria, Silvia Garattini, Mirko Barresi, Valeria Belvisi, Maria Antonella Zingaropoli, Federica Dominelli, Carmen Falvino, Fabio Mengoni, Alessandro Tavelli, Cristina Giambi, Claudio Maria Mastroianni, Cosmo Del Borgo, Raffaella Marocco, Miriam Lichtner

**Affiliations:** 1 Department of Public Health and Infectious Diseases, Sapienza University of Rome, Rome, Italy; 2 Department of NESMOS, Sapienza University of Rome, Rome, Italy; 3 Infectious Diseases Unit, SM Goretti Hospital, Latina, Italy; 4 ICONA Foundation, Milan, Italy; 5 Department of Public Health, Experimental and Forensic Medicine, National PhD Programme in One Health Approaches to Infectious Diseases and Life Science Research, University of Pavia, Pavia, Italy; 6 UOS Profilassi E Sorveglianza Malattie Infettive, SM Goretti Hospital, Latina, Italy

**Keywords:** PWH, HPV, Flow cytometry, T-cell response, Vaccination, Baseline CD4 T-cell count, 9vHPV vaccine

## Abstract

**Background:**

Most studies on HPV vaccination have focused on the antibody response and on bivalent and quadrivalent vaccines. For these reasons, an evaluation of T-cell responses in PWH on effective ART to the Gardasil 9® vaccine according to prior HPV infection and baseline CD4 T-cell count was performed.

**Methods:**

We prospectively enrolled PWH on effective ART. T-cell responses were assessed by flow cytometry at pre-vaccination (T0), on the same day of the third dose (T1) and 6 months after the third dose (T2) after stimulation with a pool of HPV16 and HPV18 L1 peptide libraries. An evaluation of IFN-γ in supernatants was also carried out. Baseline CD4 T-cell count was defined at the first dose.

**Results:**

An increase in the percentage of responding and polyfunctional T-cells was found in both HPV − and HPV + groups, with no significant differences between them at any time point. When the population was stratified according to baseline CD4 T-cell count, an increase was found only in > 500 cells/μL group for both the responding and polyfunctional T-cells. Furthermore, a higher frequency of responding CD4 T-cells at T1 (*p* = 0.0049), a higher frequency of responding CD8 T-cells at T1 and T2 (*p* = 0.05 and *p* = 0.02, respectively), a higher frequency of polyfunctional CD4 T-cells at T1 (*p* = 0.0242) and a higher frequency of polyfunctional CD8 T-cells at T2 (*p* = 0.02) were found in > 500 cells/μL group than in their relative counterpart.

The analysis of the estimated change from T0 revealed no statistically significant differences between PWH with or without prior HPV infection, whereas significant associations between higher CD4 counts and improved immune responses were found.

**Conclusions:**

Our study revealed a coordinated T-cell response against HPV16 and 18 after Gardasil9® vaccine in PWH, including those with a history of HPV infection. PWH with a lower CD4 T-cell count remains a group who may not mount a fully protective immune response and, therefore, may require additional protection or ad hoc vaccination strategies. These data reinforce the key role of basal CD4 level in modulating the immune response over time, while prior HPV infection did not significantly affect the rate and quality of the response.

**Supplementary Information:**

The online version contains supplementary material available at 10.1186/s12916-025-04504-1.

## Background

Compared with HIV-uninfected people, people with HIV (PWH) have a higher incidence and persistence of human papillomavirus (HPV) infection, leading to an augmented risk of HPV-related cancers [1–3]. Hence, a proactive approach for immunizing PWH who are susceptible to vaccine-preventable infections and at risk of exposure is needed [4, 5].

HPV prophylactic vaccines are based on recombinant protein virus-like particle (L1 VLP) with a proprietary adjuvant [6]. Currently, there are three licensed prophylactic L1 VLP-based vaccines that provide protection against two (bivalent), four (quadrivalent), and nine (nonavalent) HPV genotypes, and have demonstrated excellent safety and immunogenicity [7]. The last one, approved by the FDA in 2014, was introduced for the immunization of PWH by the Italian National Immunization Prevention Plan 2017–2019 and was reconfirmed at 2023–2025, with a three-dose schedule regardless of age [8]. The protection elicited by L1 VLP is type-restricted; therefore, to achieve broad protection, an HPV vaccine needs to contain L1 VLP of several key types. Since the most oncogenic genotypes are HPV-16 and HPV-18, all vaccines include their respective L1 VLP [9, 10].

Licensed bivalent and quadrivalent prophylactic vaccines have proven to be generally safe and well tolerated in this population [11]. Several studies on these vaccines have reported that the immune response in PWH is similar to that in the general population, with high rates of seroconversion and cellular immunogenicity comparable to that of uninfected-HIV individuals [11–15]. Furthermore, antibody levels following vaccination appear to be stable over time [16]. Significant positive correlations between T-cell responses and current CD4 T-cell count as well as negative correlations with HIV plasma RNA load have been observed, together with higher seroconversion rates among PWH with current CD4 cell counts > 200 cells/μL compared with ≤ 200 cells/μL. Furthermore a possible decline in B cell memory responses between 2 and 5 years after the last dose of vaccination has been described [17, 18].

This finding is consistent with observations from studies investigating the immunogenicity of other vaccines, but few studies on HPV vaccine immunogenicity in PWH have included participants with prior HPV infection. Furthermore, most studies have focused on the humoral response and on the immunogenicity of the bivalent and quadrivalent vaccines.

For these reasons, the aim of this study was to characterize the T-cell response to the Gardasil 9® HPV vaccine in a cohort of PWH, with a focus on its functional profile, emphasizing the potential effect of prior HPV infection and the CD4 T-cell count at the first vaccine dose.

## Methods

### Study design and population

This single-center prospective cohort study included PWH who were on ART under routine follow-up at the Infectious Diseases Unit of S.M Goretti Hospital of Latina, Italy. The participants received Gardasil 9® vaccination at months 0, 2 and 6 according to the Italian National Immunization Prevention Plan vaccination program during an initiative aimed at implementing vaccinations in adult target populations held at the SM Goretti Hospital, with an active friendly vaccination offer methodology, in the context of the Fast Track Cities network [19].

Following written informed consent, blood samples were collected from all participants at pre-vaccination (T0), on the same day of the third dose (T1) and 6 months after the third dose (T2). A flow cytometric evaluation of both responding (producing any of interferon-γ (IFN-γ), interleukin-2 (IL-2), or tumor necrosis factor α (TNFα)) and polyfunctional CD4/CD8 T-cells (producing all three cytokines), as well as an automated ELISA for IFN-γ release in supernatants, was performed on the collected samples.

Any adverse events following immunization were observed according to routine practice. Contextually, a questionnaire on sexual orientation, sexual activity and sexually transmitted infections was administered to the participants.

Participants aged < 18 and/or who had previously received quadrivalent HPV vaccination were excluded from the study. Furthermore, PWH with other active chronic viral infections (e.g., HCV, HBV) were excluded from the study population.

All enrolled PWH were stratified according to their previous history of HPV infection on the basis of anamnestic data and on the basis of their CD4 cell count at the time of the first dose into 2 groups: patients with baseline CD4 T-cell count ≤ 500 cells/µl and patients with baseline CD4 T-cell count > 500 cells/µl.

To define prior HPV infection, all available clinical documentation, including HPV DNA test results and/or cytological findings consistent with condylomatosis or HPV-related dysplastic lesions, as well as self-reported information collected prior to study enrollment was considered. Medical records reviewed dated back as early as 2003 and up to the time of enrollment.

### Assessment of HPV-specific T-cell response

Vaccine-induced T-cell responses were evaluated by an intracellular-cytokine staining flow cytometry assay after stimulation of heparinized whole blood with a pool of PepMix™ HPV16 and HPV18 L1 at a final concentration of 1 µg/ml and overnight incubation in the presence of the costimulatory antibody CD28. Specifically, PepMix™ reagents, purchased by JPT Peptide Technologies, contain 15 amino-acid peptides spanning the complete amino acid sequence of the indicated protein antigen, with an 11 amino acid overlap between adjacent peptides. For each stimulation a negative and positive (phytohemagglutinin (PHA) 5 μg/mL) condition was included. Brefeldin A at a final concentration of 5 mg/ml was added in the culture after 1 h of incubation. After overnight stimulation, 100 µl of the stimulated diluted blood was stained with an appropriate mix of conjugated antibodies. For the surface staining, PacificBlue-conjugated anti-CD45 to identify cells belonging to lymphoid and myeloid lines, APC-Cy7-conjugated anti-CD4 and APC-conjugated anti-CD8 and APC- Cy7-conjugated anti-CD4 to identify CD4 + and CD8 + T-cells, respectively, were used and incubated in darkness at 4 °C for 20 min. Then, the red blood cells were lysed using the lysing solution (BD Biosciences, Franklin Lakes, NJ, USA), in darkness at room temperature for 20 min (BD Biosciences, Franklin Lakes, NJ, USA). Fix/Perm solution (BioLegend, San Diego, CA, USA) was used prior to intracellular staining. For intracellular staining, FITC-conjugated anti-IFNγ, PerCp-Cy5.5-conjugated anti-TNFα, and PE-Cy7-conjugated anti-IL2 were used. The cells were washed once in Perm wash solution (BioLegend, San Diego, CA, USA) according to the manufacturer’s instructions. All the antibodies were from BioLegend. Finally, the cells were fixed in Phosphate-Buffered Saline (PBS) containing 0.5% formaldehyde (Sigma-Aldrich, St. Louis, MO, USA).

The stained samples were acquired using the MACSQuant Flow Cytometer (Miltenyi Biotec, Germany) and analyzed using FlowJoTM v10.8.1 software, and the cytokine background obtained from the negative condition (unstimulated) was subtracted from the stimulated ones. Co-expression of cytokines was analyzed via Boolean gating using FlowJoTM v10.8.1. We identified T-cells producing any of IFN-γ, IL2 or TNFα as responding T-cells and polyfunctional T-cells as those simultaneously producing all 3 cytokines, as previously mentioned.

An evaluation of IFN-γ in supernatants has also been carried out using an automated ELISA (ELLA, Protein Simple), following the manufacturer’s instructions. Briefly, 500 μl of fresh heparinized blood were incubated overnight after stimulation. For each stimulation, a negative and positive (PHA 5 μg/mL) condition were included. After 18 h, supernatants were collected and stored at − 80 °C until use. The detection limit of these assays was 0.17 pg/mL. The response has been defined as the peptide or PHA stimulated condition minus the unstimulated condition.

### Statistical analysis

Descriptive statistics are presented as median with interquartile range (IQR) for continuous variables and frequencies with proportion for categorical variables. To investigate the effects of the baseline CD4 T-cell count (≤ 500 vs > 500 CD4/μl) and prior HPV infection on the magnitude of immune response to Gardasil 9® vaccination at different timepoints, for the unadjusted comparison within each group, parameters at T1 and T2 were compared with the baseline level using the paired Wilcoxon sign-rank test. For the comparison between groups, the Kruskal–Wallis test was performed to determine if groups were significantly different on all continuous variables considered. Specifically, the Dunn test with Bonferroni correction was used for pairwise multiple comparisons of each parameter between any pairs.

Adjusted linear mixed models with random intercept and slope for individual and robust standard errors, were also used to estimate mean levels of CD4 − and CD8 − responding and polyfunctional cells as well as the mean IFN-γ levels at the three timepoints, which were then compared across exposure groups of baselines CD4 T-cell count (> 500 vs ≤ 500 CD4/µl) and previous HPV infection (Yes vs No). The models were adjusted for age, sex, CD4 nadir and HIV viral load suppression at baseline and baseline CD4 or prior HPV as appropriate. The estimated mean changes from T1 to T0 and T2 to T0 between groups of interest (CD4 > 500 vs ≤ 500 cells/µl and previous HPV infection vs No) together with 95% CI are also reported.

A post hoc power analysis for two independent means with different group sizes (*n* = 9 for the CD4 ≤ 500/μL group and *n* = 29 for the CD4 > 500/μL group) was also conducted. Power was calculated to detect the observed (unadjusted) mean differences in the changes of immunological response from T0 to T1 and T2 (ΔT1–T0 and ΔT2–T0) between the two CD4 strata, using pooled standard deviations estimated from the changes for each marker. The analysis was performed retrospectively to assess whether the sample size was adequate to detect the differences we observed between CD4 strata. A 2-sided *P* value < 0.05 was considered statistically significant. Analyses were performed using STATA v18.0.

Distribution differences of the different cytokine combinations between stratified groups were performed with a nonparametric permutation test and Wilcoxon signed-rank test, using SPICE, distributed by the National Institute of Allergy and Infectious Diseases, NIH.

All figures were created using GraphPad Prism v.9.

## Results

### Characteristics of the study population

A total of 38 PWH with a median age (IQR) of 41.0 (34.0–50.0) and a median time elapsed from diagnosis (IQR) of 8.5 (3–12) years, in good immune-virological status were included in the study between September 2022 and July 2023. Their main characteristics are shown in Table 1.

**Table 1:** Demographic characteristics of the study population

	**PWH**
***N***	**38**
**Sex, *n* (%)**	
M	29 (76.3%)
F	9 (23.7%)
**Age, median (IQR)**	41.0 (34.0–50.0)
**Years from HIV diagnosis, median (IQR)**	8.5 (3.5–12.0)
**Calendar year 1st vaccine dose, *n* (%)**	
2022	15 (39.5%)
2023	23 (60.5%)
**CD4 count nadir, cells/µL, median (IQR)**	320.5 (105.0–552.0)
< 200 cells/µL, *n* (%)	11 (32.4%)
**Baseline CD4 count, cells/µL, median (IQR)**	716.0 (518.0–881.0)
≤ 500 CD4/µL, *n* (%)	9 (24.0%)
> 500 CD4/µL, *n* (%)	29 (76.0%)
**Baseline HIV-RNA, cps/ml**	
< 50 cps/ml, *n* (%)	34 (92.0%)
≥ 50 cps/ml, *n* (%)	3 (8.0%)
**ART class, *n* (%)**	
2DR	14 (36.8%)
3DR	23 (60.5%)
Other	1 (2.6%)
**Comorbidities, *n* (%)**	
No	28 (73.7%)
Yes	10 (26.3%)
**CMV-IgG, *n* (%)**	
Negative	3 (7.9%)
Positive	35 (92.1%)
**History of HPV infection, *n* (%)**	13 (34.2%)
**Smoking, *n* (%)**	
Unknown	3 (8.0%)
No	12 (32.0%)
Yes	23 (60.0%)
**STI previous 6 months, *n* (%)**	
Unknown	2 (5.3%)
No	34 (89.4%)
Yes	2 (5.3%)
**Sexual orientation, *n* (%)**	
MSM	24 (63.0%)
Bisex	1 (3.0%)
MSW/WSM	13 (34.0%)
**partner last 6 months, *n* (%)**	
Unknown	13 (34.0%)
7–10	2 (5.0%)
4–6	1 (4.0%)
1–3	15 (39.0%)
0	7 (18.0%)

Interquartile range (IQR) or *n*. (%) of subjects

*Abbreviations*: *PWH* people with human immunodeficiency virus, *M* male, *F* female, *ART* antiretroviral treatment, *2DR* two-drug regimen, *3DR* three-drug regimen, *CMV* cytomegalovirus, *HPV* human papillomavirus, *STI* sexually transmitted infections, *MSM* men who have sex with men, *MSW* men who have sex with women, *WSM* women who have sex with men

Nadir and baseline median CD4 T-cell count (IQR) of PWH were 320.5 (105.0–552.0) cells/μL and 716.0 (518.0–881.0) cells/μL, respectively. Nine of them (24%) had a baseline CD4 T-cell count ≤ 500 cell/μL. For three of the 38 participants (8%) HIV-RNA was detectable (< 80 cp/mL). All PWH were on ART at the time of vaccination. Specifically, 36.8% of them were on a dual ART regimen, while 60.5% were on a 3-drug ART regimen. Most of the participants (73.7%) had no comorbidities, no one was on immunosuppressive regimens for concomitant pathologies, and 92.1% had detectable levels of anti-CMV IgG. According to anamnestic data, 34.2% of PWH had a history of HPV infection. HPV test results and/or cytological findings were available for 8 of the 13 individuals: 2 women (for whom only HPV DNA test results were available) and 6 men. The reported genotypes included HPV 33 in one case, HPV 16 in two cases (one of whom was also diagnosed with atypical squamous cells of undetermined significance (ASCUS)), and a coinfection with HPV types 33, 35, 39, 45, 51, and 52 in one case, which was also associated with clinically documented anal warts. Based on cytological findings, one man was diagnosed with ASCUS, two (both men) with low-grade squamous intraepithelial lesion (LSIL) and one man with anal warts. In these patients, no HPV DNA sample was available at the time of the study.

The baseline characteristics of PWH stratified according to CD4 T-cell count (> 500 vs ≤ 500 CD4/µl) and previous HPV infection (Yes vs No) are reported in Additional file 1: Table 1.

### Frequency of cytokine response, polyfunctionality and IFN-γ release

**Fig. 1:**
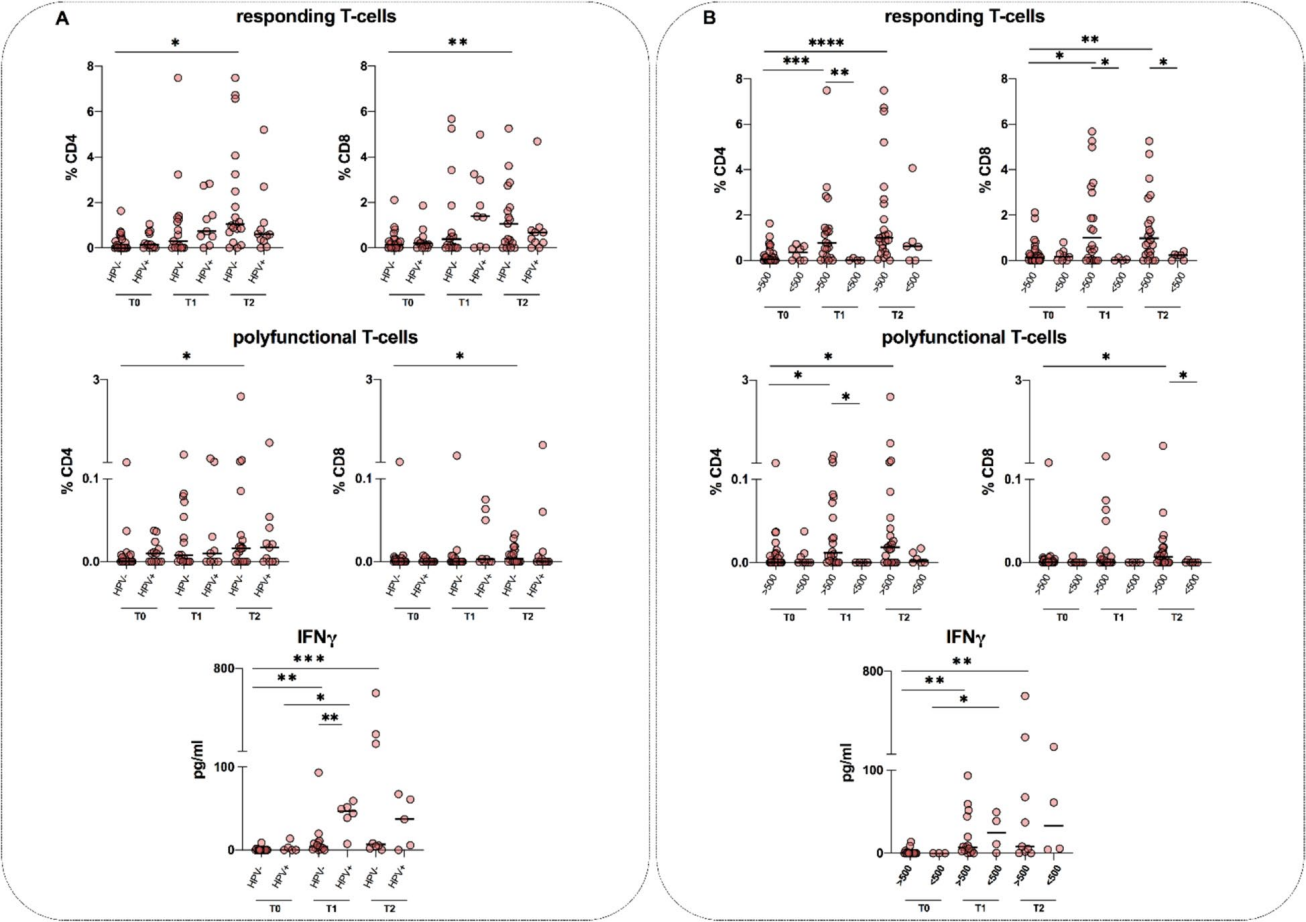
Evaluation of the frequency of cytokine response, polyfunctionality and IFN-γ release to L1 of HPV16 and HPV18 in PWH stratified according to HPV infection (**A**) and CD4 T-cell count (**B**). The horizontal lines represent the median. Statistical analysis was performed using Dunn’s multiple comparison test, and the *p* value was considered significant if < 0.05. Statistical significance (*p*) is reported in the graphics. ****p < 0.0001; *** *p* < 0.001; ** *p* < 0.01; *p < 0.05. Pre-vaccination (T0), on the same day of the third dose (T1) and 6 months after the third dose (T2)

When our population was stratified according to prior HPV infection, an increase in the frequency of responding CD4 and CD8 T-cells was found at T2 compared with T0 (*p* = 0.088 and *p* = 0.0064, respectively) in the HPV − group, as well as in the HPV + group; although in this group, this increase was not significant (Fig. 1A). Comparing the two subgroups, no differences in the frequency of the responding T-cells were found at any time (Fig. 1A). Concerning the polyfunctional subset, an increase in the frequency of the polyfunctional CD4 and CD8 T-cell compartment was found at T2 compared to T0 in the HPV − group (*p* = 0.0255 and *p* = 0.05, respectively), as well as in the HPV + group. When the two subgroups were compared, no differences in the frequency of the polyfunctional T-cells were found at any time (Fig. 1A). In terms of IFN-γ release, a higher concentration of this cytokine was detected at T1 in those subjects with prior HPV infection compared with those with no prior HPV infection (*p* = 0.0092) (Fig. 1A).

**Fig. 2:**
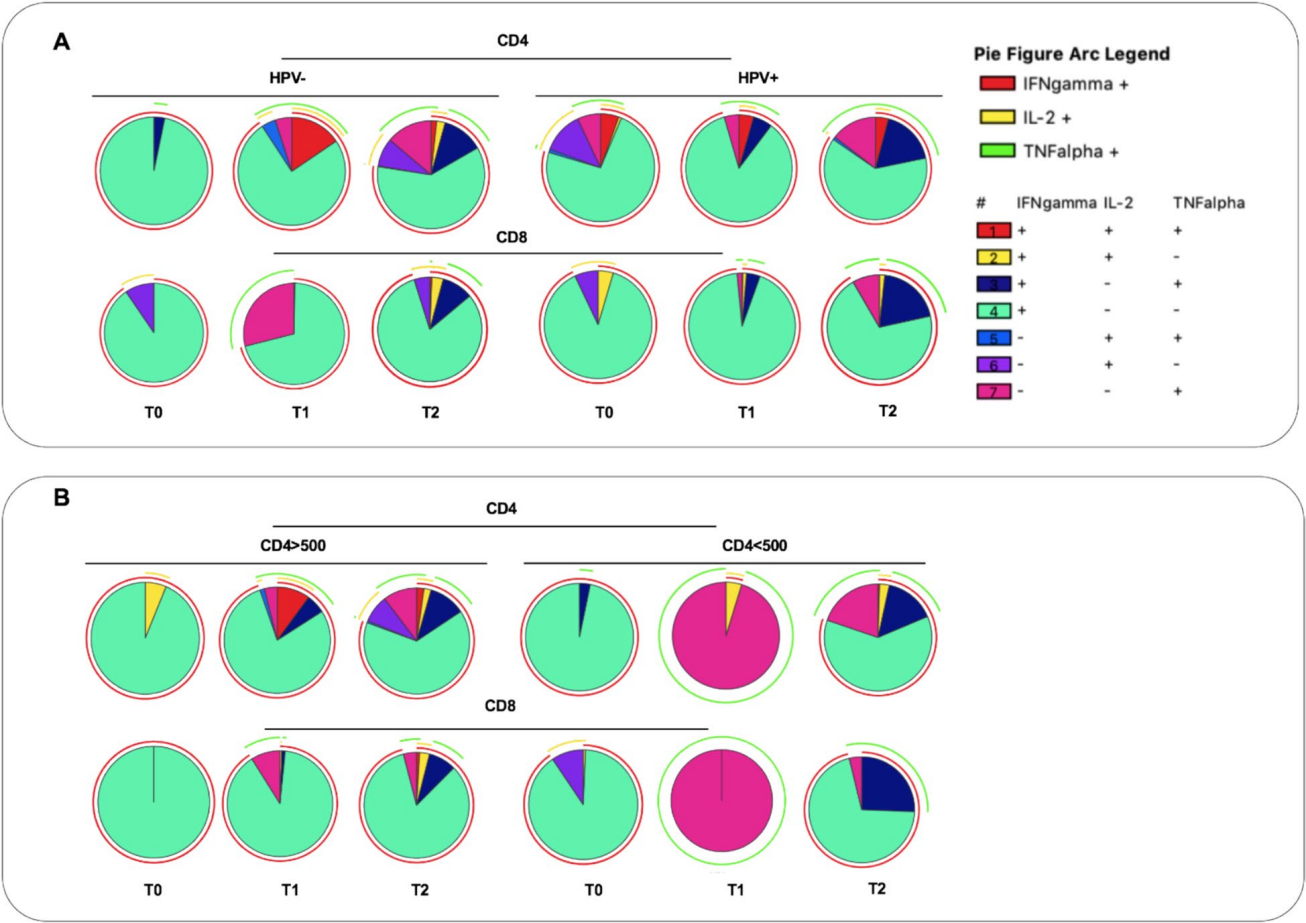
Frequency of CD4 and CD8 T cells producing all combinations of IFN-γ, IL2, and TNFα in PWH stratified according to HPV infection (**A**) and CD4 cell count (**B**). Differences in the distribution of the cytokine combinations between stratified groups were analyzed via a nonparametric permutation test. Pre-vaccination (T0), on the same day of the third dose (T1) and 6 months after the third dose (T2)

Stratifying our population according to the baseline CD4 T-cell count into those with a cell count ≤ 500 cells/μL and those who have it above, a significant increase in the frequency of both responding CD4 and CD8 T-cells was found at T1 and T2 compared with T0 (CD4: *p* = 0.0008 and *p* < 0.0001, respectively; CD8: *p* = 0.0134 and *p* = 0.0024, respectively) only in the > 500 cells/μL subgroup (Fig. 1B). Comparing the two subgroups, a higher frequency of responding CD4 T-cells was found at T1 in the > 500 cells/μL group (*p* = 0.0049), along with a higher frequency of responding CD8 T-cells at T1 and T2 (*p* = 0.05 and *p* = 0.02, respectively) (Fig. 1B). Concerning the polyfunctional subset, an increase in the frequency of the polyfunctional CD4 T-cell compartment was found at T1 and T2 compared to T0 only in the > 500 cells/μL group (*p* = 0.0226 and *p* = 0.0155 respectively), as well as an increase in the frequency of the polyfunctional CD8 T-cell compartment at T2 compared to T0 (*p* = 0.0181) (Fig. 1B). Furthermore, when the two subgroups were compared, a higher frequency of polyfunctional CD4 T-cells at T1 (*p* = 0.0242), along with a higher frequency of polyfunctional CD8 T-cells at T2 (*p* = 0.02) was found in the > 500 cells/μL subgroup (Fig. 1B). No statistically significant differences were found between those with a baseline CD4 T-cell count ≤ 500 cells/μL and their relative counterpart concerning IFN-γ release (Fig. 2B). All medians and IQR are reported in Additional file 2: Table 1.

### Role of prior HPV infection and baseline CD4 T-cell count in predicting immunogenicity in the adjusted analysis

Overall, no statistically significant differences were observed between PWH with and without prior HPV infection across all parameters and time points (Table 2, left).

**Table 2:** Estimated changes from T0 in PWH stratified according to prior HPV infection and baseline CD4 T-cell count

		Estimated mean change from T0 (95%CI)	Δ estimated change from T0 in PWH with prior HPV infection vs no prior infection (95%CI)	*p*-value			Estimated mean change from T0 (95%CI)	Δ estimated change from T0 in > 500 CD4/µl vs ≤ 500 CD4/µl (95%CI)	*p*-value
**CD4-responding**									
**T1**	**HPV + **	+ 0.854 (− 0.276, + 1.984)	− 0.053 (− 1.334, + 1.227)	0.935	**T1**	**CD4 ≤ 500 cells/µl**	− 0.187 (− 0.504, + 0.130)	+ 1.292 (+ 0.363, + 2.221)	0.006
	**HPV − **	+ 0.801 (+ 0.215, + 1.387)				**CD4 > 500 cells/µl**	+ 1.104 (+ 0.224, + 1.985)		
**T2**	**HPV + **	+ 1.920 (+ 0.823, + 3.012)	− 1.106 (− 2.530, + 0.318)	0.128	**T2**	**CD4 ≤ 500 cells/µl**	+ 0.638 (− 0.423, + 1.699)	+ 1.144 (− 0.280, + 2.567)	0.115
	**HPV − **	+ 0.814 (− 0.098, + 1.726)				**CD4 > 500 cells/µl**	+ 1.782 (+ 0.833, + 2.731)		
**CD4-polyfunctional**									
**T1**	**HPV + **	+ 0.037 (− 0.011, + 0.085)	− 0.00 (− 0.073, + 0.074)	0.944	**T1**	**CD4 ≤ 500 cells/µl**	− 0.008 (− 0.015, − 0.001)	+ 0.057 (+ 0.012, + 0.102)	0.013
	**HPV − **	+ 0.037 (− 0.018, + 0.092)				**CD4 > 500 cells/µl**	+ 0.049 (+ 0.004, + 0.093)		
**T2**	**HPV + **	+ 0.148 (− 0.097, + 0.392)	− 0.072 (− 0.352, + 0.208)	0.613	**T2**	**CD4 ≤ 500 cells/µl**	+ 0.002 (− 0.011, + 0.016)	+ 0.155 (− 0.056, + 0.366)	0.151
	**HPV − **	+ 0.076 (− 0.061, + 0.212)				**CD4 > 500 cells/µl**	+ 0.157 (− 0.053, + 0.368)		
**CD8-responding**									
**T1**	**HPV + **	+ 0.704 (− 0.206, + 1.615)	+ 0.662 (− 0.732, + 2.056)	0.352	**T1**	**CD4 ≤ 500 cells/µl**	− 0.264 (− 0.428, − 0.101)	+ 1.569 (+ 0.745, + 2.394)	0.01
	**HPV − **	+ 1.367 (+ 0.321, + 2.413)				**CD4 > 500 cells/µl**	+ 1.305 (+ 0.482, + 2.128)		
**T2**	**HPV + **	+ 1.720 (+ 0.305, + 3.136)	+ 0.270 (− 2.955, + 3.495)	0.87	**T2**	**CD4 ≤ 500 cells/µl**	+ 0.048 (− 0.234, + 0.330)	+ 2.339 (− 0.532, + 4.145)	0.011
	**HPV − **	+ 1.991 (− 0.903, + 4.885)				**CD4 > 500 cells/µl**	+ 2.386 (+ 0.599, + 4.174)		
**CD8-polyfunctional**									
**T1**	**HPV + **	+ 0.036 (− 0.019, − 0.087)	− 0.012 (− 0.069, + 0.046)	0.686	**T1**	**CD4 ≤ 500 cells/µl**	− 0.001 (− 0.004, + 0.002)	+ 0.038 (− 0.004, + 0.080)	0.078
	**HPV − **	+ 0.022 (+ 0.001, + 0.041)				**CD4 > 500 cells/µl**	+ 0.037 (− 0.005, + 0.079)		
**T2**	**HPV + **	+ 0.172 (− 0.158, + 0.502)	− 0.102 (− 0.454, + 0.245)	0.57	**T2**	**CD4 ≤ 500 cells/µl**	+ 0.008 (− 0.008, + 0.024)	+ 0.165 (− 0.111, + 0.441)	0.241
	**HPV − **	+ 0.070 (− 0.051, + 0.191)				**CD4 > 500 cells/µl**	+ 0.173 (− 0.103, + 0.445)		
**IFN-γ, pg/mL**									
**T1**	**HPV + **	+ 16.088 (+ 1.584, + 30.592)	+ 11.157 (− 11.458, + 33.773)	0.334	**T1**	**CD4 ≤ 500 cells/µl**	+ 16.106 (+ 1.245, + 30.968)	+ 5.525 (− 15.496, + 26.545)	0.606
	**HPV − **	+ 27.245 (+ 9.840, + 44.650)				**CD4 > 500 cells/µl**	+ 21.631 (+ 6.678, + 36.584)		
**T2**	**HPV + **	+ 134.521 (+ 1.081, + 267.962)	− 114.316 (− 249.654, + 21.022)	0.098	**T2**	**CD4 ≤ 500 cells/µl**	+ 74.605 (− 2.054, + 151.264)	+ 20.703 (− 115.543, + 156.950)	0.766
	**HPV − **	+ 20.206 (− 1.270, + 41.681)				**CD4 > 500 cells/µl**	+ 95.308 (− 16.859, + 207.476)		

Adjusted for age, sex, CD4 nadir, and HIV viral load suppression at baseline and baseline CD4 or prior HPV as appropriate

*CI* confidence interval, *PWH* people with human immunodeficiency virus, *HPV* human papilloma virus

Conversely, when the population was stratified according to baseline CD4 T-cell count, significant associations between higher CD4 counts and improved immune responses were found (Table 2, right). Specifically, individuals with CD4 > 500 cells/μL had a higher mean increase in the percentage of responding CD4 T-cells at T1 (Δ T1–T0 in > 500 vs ≤ 500 CD4/μL = + 1.292; 95% CI 0.363, 2.221; *p* = 0.006) and T2 (Δ T2–T0 in > 500 vs ≤ 500 CD4/μL = 1.144; 95%CI − 0.280, + 2.567; *p* = 0.115) compared to those with CD4 ≤ 500 cells/μL (Table 2, right). The same result was found concerning polyfunctional CD4 T-cells, with a higher mean increase at T1 in the > 500 CD4 group (Δ T1–T0 in > 500 vs ≤ 500 CD4/μL = + 0.057; 95%CI: 0.012, 0.102; *p* = 0.013) and marginally at T2 (Δ T2–T0 in > 500 vs ≤ 500 CD4/μL = + 0.155; 95%CI − 0.056, + 0.366; *p* = 0.151) (Table 2, right). Furthermore, the > 500 group exhibited a significantly higher increase in the responding CD8 T-cells subset at both T1 and T2 compared with ≤ 500 CD4 cells/μL (Δ T1–T0 in > 500 vs ≤ 500 CD4/μL = + 1.569; 95%CI: + 0.745, + 2.394; *p* = 0.01; Δ T2–T0 in > 500 vs ≤ 500 CD4/μL = + 2.339; 95% CI: − 0.532; + 4.145; *p* = 0.011). The mean increases in the percentage of the polyfunctional CD8 T-cell subset in the > 500 CD4/ μL group were + 0.037 (95% CI: − 0.005, + 0.079), with a difference compared to ≤ 500 CD4/μL of + 0.038 (95% CI: − 0.004; + 0.080) that did not reach statistical significance (*p* = 0.078), and a similar trend was observed, with a Δ of + 0.165 95%CI (*p* = 0.241), at T2. Concerning IFN-γ levels comparisons, the differences were not statistically significant (Table 2, right).

To evaluate the impact of the limited sample size of the CD4 ≤ 500 cells/μL group (*n* = 9), a post hoc power analysis was conducted based on observed mean differences and standard deviations. Despite the small number of participants, the power was > 80% for key comparisons including CD4-polyfunctional, CD8-responding, and CD8-polyfunctional responses at T2. However, the power was limited (< 60%) for several other comparisons (Additional File 3: Table 1).

### Functional profile of T-cell responses according to prior HPV infection and baseline CD4-T-cell count

Looking at the functional cytokine profile in PWH stratified according to prior HPV infection, a heterogeneous distribution of all the CD4 and CD8 subpopulations was found at T1 and T2 in both groups and, when the functional cytokine profiles were compared between them, no differences were found at all time points (Fig. 2A).

Concerning the second exposure of interest, a heterogeneous distribution of all the CD4 and CD8 subpopulations was found at both T1 and T2 in the > 500 cells/μL group, whereas in the relative counterpart, the response showed a different dynamic. Specifically, at T1, a statistically significant difference in the profile of the CD4 T-cell response was found (*p* = 0.0176), with a higher frequency of CD4 polyfunctional T-cells (*p* = 0.0201) and CD4 IFN-γ producing T-cells (*p* = 0.0332) in the > 500 cells/μL group, than in the relative counterpart, which instead showed a predominantly monofunctional response (Fig. 2B). The same result was observed for CD8 T-cells (*p* = 0.0155). At T2, no differences were found in the functional profile of the response between the two groups. However, higher percentages of polyfunctional CD4 and CD8 T-cells (*p* = 0.09 and *p* = 0.0406, respectively) were observed in the > 500 cells/μL group compared with the relative counterpart.

## Discussion

In the present study, we aimed at implementing the knowledge on the T-cell response in PWH to the Gardasil 9® vaccine, with a focus on the dynamics of its functional profile, emphasizing the potential effects of prior HPV infection and the CD4 T-cell count at the first dose of vaccination.

Specifically, we evaluated T-cell responses in a population of PWH receiving effective ART before the first dose was administered, at the time of administration of the third dose and at 6 months after the third dose, according to prior history of HPV infection, based on anamnestic data, and baseline CD4 T-cell count.

We decided to include in our study PWH with a previous history of HPV infection, which are usually excluded from the design of many studies, in order to evaluate the possible effects on the T-cell response. Vaccination with L1 VLP does not confer a therapeutic benefit in most disease models or clinical studies, but people with prior infection can, in any case, benefit from vaccination [20]. In fact, prevalent infection by one type does not impede vaccine-induced protection from incident infection by another vaccine type and antibodies induced by vaccination are higher than those induced by natural infection, protecting individuals from reinfection [21]. Furthermore, beneficial effects on the recurrence of high-grade anal neoplasia have also been reported, suggesting the utility of vaccination not only in primary prevention, but also as an effective post-treatment adjuvant form of therapy [22, 23]. Our results support, from an immunological point of view, that previously infected PWH may benefit from vaccination. In fact, an increase in the frequency of both responding and polyfunctional T-cells was found, along with an improvement in the quality of the response, particularly after the completion of the cycle. Specifically, the subset distribution of CD4 T-cells suggested a Th1 polarization of antigen-specific CD4 T-cells, supporting a cellular immune response that is effective in viral clearance and long-term protection. Similarly, the subset distribution of CD8 T-cells was in line with the CTL activity explicated by these cells, which is consistent with other studies [24–26]. These features are crucial in the context of HIV-related immune dysregulation, where impaired T-cell function can limit vaccine efficacy. Concerning the evaluation of extracellular cytokines, a higher concentration of IFN-γ was found at T1 in those subjects with a history of prior HPV infection than in those without a history of prior HPV infection, which is compatible with the fact that their cells may be primed by natural infection [26]. The observed IFN-γ response could potentially also be attributed to infection with multiple genotypes that “synergize” in inducing cytokine production [27].

The difference observed in the data obtained from intracellular staining aligns with the fact that this technique allows for the identification of all producing cells, whereas the extracellular evaluation does not provide any information regarding this.

Our cohort did not include PWH with severe immune impairment. However, we decided to stratify the population according to baseline CD4 T-cell count into those with a baseline CD4 cell count above and under 500 cell/µl. Overall, we found that the third dose improved both the magnitude and the quality of the response particularly in the group with baseline CD4 T-cell count ≤ 500 cell/µl. In fact, before the administration of the third dose, they showed a lower percentage of responding T-cells compared with the relative counterpart. Concerning the functional profile of the T-cell response, a particular signature, already described in other settings, characterized by the prevalence of monofunctional T-cells producing TNF-α, was observed at T1 [28–30]. Among these patients, only one had a detectable viremia (80 cp/mL) and, collectively, the small number of patients with a detectable viremia did not allow us to assess its effects on the immune response [31]. A monofunctional T-cell response may reflect a less effective or less durable immune response compared to a polyfunctional T-cell profile, which is often associated with superior protection and long-term immunity. Specifically, monofunctional T-cells producing TNF-α have been associated with less efficient viral clearance and disease severity in other infectious contexts. Future studies should investigate whether this functional profile persists over time and whether it correlates with clinical outcomes such as protection from infection. Moreover, understanding the mechanisms driving this functional polarization should be further explored.

We do not exclude that in PWH with optimal immune reconstitution, the first two doses were sufficient to achieve full T-cell immunization, as observed for other vaccinations [32–34]. In any case, improvements in both the magnitude and the quality of the response after the third dose were also observed in this category of people.

Our study confirms that the vaccine is able to elicit a broad and coordinated immune response in our population, the benefits that people with a history of HPV infection might receive from vaccination and, finally, that PWH with a lower baseline CD4 T-cell count at first dose administration remains a group who may not mount a fully protective immune response and, therefore, may require additional protection or ad hoc vaccination strategies.

Furthermore, it confirms a key role of the basal CD4 level in modulating the immune response in time, supporting the importance of this parameter in prognosis and clinical management [35, 36]. Conversely, in our experience, prior HPV infection did not significantly affect the immunological parameters over time, consistent with the fact that once the T-response is primed, the magnitude of that response does not change significantly with subsequent immunizations [26, 37].

Even if serum neutralizing antibodies are thought to be the major protective branch of adaptive immunity afforded by L1 VLP-based vaccines, T-cell mediated immunity is critical for the defense against infections as well as for the long-term vaccine-induced response [38, 39]. Memory lymphocyte-mediated protection may persist despite low serum antibody titers, as shown for the hepatitis B vaccine and this is especially significant for certain populations, such as PWH, who may not develop as strong antibody responses but can still benefit from long-term protection via T-cell response [39].

There are some limitations in this study. A formal sample size calculation was not conducted prior to PWH enrolment due to the exploratory nature of the study and the absence of preliminary data on T-cell responses to this vaccination in our subgroups. Therefore, we have to consider that the relatively small number of participants in each group may affect the reliability of the results. This study was conducted within the framework of an initiative aimed at promoting vaccination in adult target populations, using an active and friendly vaccination offer approach. Participants were balanced in terms of gender, age, and availability of biological samples at all three time points. Importantly, the study includes individuals with a history of prior HPV infection, a group that is often underrepresented in similar research. However, we acknowledge that the small size of the CD4 ≤ 500 subgroup may have limited our ability to detect smaller differences across markers and timepoints, which should be considered when interpreting null results. In fact, the post hoc power analysis revealed sufficient power (> 80%) only for selected endpoints such as CD4‑polyfunctional and CD8‑responding outcomes at T2, while other comparisons showed limited power. Nevertheless, we have evidence for significant differences between CD4 strata for several key markers, indicating that these effects were robust enough to be identified despite the limited power. The classification of prior HPV infection was based on anamnestic data, which may introduce recall bias, and no additional diagnostic methods, such as serological testing, were used. However, these are known to have limitations in this context, as many individuals do not develop a detectable antibody response to HPV. Male Caucasian adults are the most represented, poorly reflecting the global prevalence of PWH. We did not include a healthy control group, although we speculate that the response is similar, in terms of magnitude, to that described in PWH with an optimal immune restoration, although with possible differences in the dynamics of the response. Finally, the follow-up in our study was limited to determine the duration of the immunity, although bivalent and quadrivalent vaccines have been reported to be long-lasting and more effective than natural infection in uninfected people [39, 40].

It is important to underpin that the immune system operates primarily within tissue microenvironments under physiological and pathological conditions and that only a small fraction of all T-cells circulate in the peripheral blood at any given time. Therefore, the understanding of anatomically localized T cells to inform the development of more effective immunotherapies and vaccines is needed and as sampling methods and single-cell technologies continue to evolve, this goal becomes eminently more achievable [41, 42].

In our opinion, it is possible to draw some general conclusions. Specific subgroups of PWH can benefit from ad hoc immunization strategies, such as an adapted vaccine schedule, additional doses or use of adjuvants to improve immunological responses to achieve a proper level of immunization and, in this regard, the analysis of the functional profile of the T-cell response can represent a useful tool to capture some aspects of the immune response that may help guide us through this process.

## Conclusions

Our study confirms that the vaccine is able to elicit a broad and coordinated immune response in PWH. People with a history of HPV infection may still benefit from vaccination. However, those with a lower baseline CD4 T-cell count at the first dose represent a subgroup who may not mount a fully protective immune response. A deeper understanding of the immune response in PWH may support the development of personalized vaccination strategies, based not only on demographic or clinical criteria, but also on functional immunological profiles. These approaches, combined with integrated prevention and screening policies, could significantly improve long-term outcomes and reduce the HPV-related cancer burden in this population.

## Supplementary Information

Additional file 1: Table 1. [Demographic and clinical characteristics at baseline according to prior HPV infection and CD4 T-cell count at first HPV vaccine dose].

Additional file 2: Table 1. [Medians and IQR of immunological parameters in PWH stratified according to prior HPV infection and CD4 T-cell count].

Additional file 3: Table 1. [Post-hoc power analysis on changes on immune response according to CD4 strata].

## Data Availability

The datasets used and/or analysed during the current study are available from the corresponding author on reasonable request.

## Abbreviations

PWH: People with HIV
HPV: Human papillomavirus
ART: Antiretroviral therapy/treatment
CD4: Cluster of differentiation 4 (T-cell subset)
T0, T1, T2: Pre-vaccination (T0), on the day of the third dose (T1), 6 months after the third dose (T2)
IFN-γ: Interferon gamma
IL-2: Interleukin 2
TNFα: Tumor necrosis factor alpha
L1 VLP: L1 virus-like particle
FDA: Food and Drug Administration
ASCUS: Atypical squamous cells of undetermined significance
LSIL: Low-grade squamous intraepithelial lesion
2DR: Two-drug regimen
3DR: Three-drug regimen
CMV: Cytomegalovirus
STI: Sexually transmitted infections
MSM: Men who have sex with men
MSW: Men who have sex with women
WSM: Women who have sex with men
PHA: Phytohemagglutinin
ELISA: Enzyme-linked immunosorbent assay
PBS: Phosphate buffered saline
IQR: Interquartile range
CI: Confidence interval

## References

[1] Poljak M, Šterbenc A, Lunar MM. Prevention of human papillomavirus (HPV)-related tumors in people living with human immunodeficiency virus (HIV). Expert Rev Anti-Infect Ther. 2017;15:987–99. 29027811 10.1080/14787210.2017.1392854

[2] Park LS, Hernández-Ramírez RU, Silverberg MJ, et al. Prevalence of non-HIV cancer risk factors in persons living with HIV/AIDS: a meta-analysis. AIDS Lond Engl. 2016;30:273–91. 10.1097/QAD.0000000000000922 PMC4689318 26691548

[3] Machalek DA, Poynten M, Jin F, et al. Anal human papillomavirus infection and associated neoplastic lesions in men who have sex with men: a systematic review and meta-analysis. Lancet Oncol. 2012;13:487–500. 22445259 10.1016/S1470-2045(12)70080-3

[4] CDC. CDC Works 24/7. Centers for Disease Control and Prevention. 2024. https://www.cdc.gov/index.htm. Accessed 26 Mar 2024.

[5] Geretti AM, BHIVA Immunization Writing Committee, Brook G, et al. British HIV Association guidelines for immunization of HIV-infected adults 2008. HIV Med. 2008;9:795–848. 10.1111/j.1468-1293.2008.00637.x 18983477

[6] Schiller JT, Castellsagué X, Villa LL, et al. An update of prophylactic human papillomavirus L1 virus-like particle vaccine clinical trial results. Vaccine. 2008;26:K53–61. 18847557 10.1016/j.vaccine.2008.06.002 PMC2631230

[7] Aggarwal S, Agarwal P, Singh AK. Human papilloma virus vaccines: a comprehensive narrative review. Cancer Treat Res Commun. 2023;37:100780. 38006748 10.1016/j.ctarc.2023.100780

[8] Salute M della. Piano nazionale prevenzione vaccinale. https://www.salute.gov.it/portale/vaccinazioni/dettaglioContenutiVaccinazioni.jsp?lingua=italiano&id=4828&area=vaccinazioni&menu=vuoto. Accessed 13 Jul 2023.

[9] Roden RBS, Stern PL. Opportunities and challenges for human papillomavirus vaccination in cancer. Nat Rev Cancer. 2018;18:240–54. 29497146 10.1038/nrc.2018.13 PMC6454884

[10] Schiller JT, Müller M. Next generation prophylactic human papillomavirus vaccines. Lancet Oncol. 2015;16:e217–25. 25943066 10.1016/S1470-2045(14)71179-9

[11] Giacomet V, Penagini F, Trabattoni D, et al. Safety and immunogenicity of a quadrivalent human papillomavirus vaccine in HIV-infected and HIV-negative adolescents and young adults. Vaccine. 2014;32:5657–61. 25149430 10.1016/j.vaccine.2014.08.011

[12] Denny L, Hendricks B, Gordon C, et al. Safety and immunogenicity of the HPV-16/18 AS04-adjuvanted vaccine in HIV-positive women in South Africa: a partially-blind randomised placebo-controlled study. Vaccine. 2013;31:5745–53. 24091311 10.1016/j.vaccine.2013.09.032

[13] Pinto LA, Wilkin TJ, Kemp TJ, et al. Oral and systemic HPV antibody kinetics post-vaccination among HIV-positive and HIV-negative men. Vaccine. 2019;37:2502–10. 30940485 10.1016/j.vaccine.2019.03.034 PMC6863043

[14] Wilkin T, Lee JY, Lensing SY, et al. Safety and immunogenicity of the quadrivalent human papillomavirus vaccine in HIV-1–infected men. J Infect Dis. 2010;202:1246–53. 20812850 10.1086/656320 PMC3118428

[15] Zurek Munk-Madsen M, Toft L, Kube T, et al. Cellular immunogenicity of human papillomavirus vaccines Cervarix and Gardasil in adults with HIV infection. Hum Vaccin Immunother. 2017;14:909–16. 29172992 10.1080/21645515.2017.1407896 PMC5893199

[16] Chow EPF, Fairley CK, Zou H, et al. Human papillomavirus antibody levels following vaccination or natural infection among young men who have sex with men. Clin Infect Dis. 2022;75:323–9. 34971362 10.1093/cid/ciab1052

[17] Weinberg A, Huang S, Moscicki A-B, et al. Persistence of memory B-cell and T-cell responses to the quadrivalent HPV vaccine in HIV-infected children. AIDS. 2018;32:851–60. 29424778 10.1097/QAD.0000000000001773 PMC5869173

[18] Kojic EM, Kang M, Cespedes MS, et al. Immunogenicity and safety of the quadrivalent human papillomavirus vaccine in HIV-1-infected women. Clin Infect Dis. 2014;59:127–35. 24723284 10.1093/cid/ciu238 PMC4305143

[19] Benvenuta/o a Latina Checkpoint. Latina Checkpoint. https://www.latinacheckpoint.it/. Accessed 31 Dec 2024.

[20] Jardine D, Lu J, Pang J, et al. A randomized trial of immunotherapy for persistent genital warts. Hum Vaccines Immunother. 2012;8:623–9. 10.4161/hv.19319 PMC3495719 22634446

[21] Schiller JT, Castellsagué X, Garland SM. A review of clinical trials of human papillomavirus prophylactic vaccines. Vaccine. 2012;30:F123–38. 23199956 10.1016/j.vaccine.2012.04.108 PMC4636904

[22] Swedish KA, Factor SH, Goldstone SE. Prevention of recurrent high-grade anal neoplasia with quadrivalent human papillomavirus vaccination of men who have sex with men: a nonconcurrent cohort study. Clin Infect Dis Off Publ Infect Dis Soc Am. 2012;54:891–8. 10.1093/cid/cir1036 22291111

[23] Ciccarese G, Herzum A, Serviddio G, et al. Efficacy of human papillomavirus vaccines for recalcitrant anogenital and oral warts. J Clin Med. 2023;12:7317. 38068369 10.3390/jcm12237317 PMC10706929

[24] McElhaney JE, Gravenstein S, Upshaw CM, et al. Immune response to influenza vaccination in institutionalized elderly: effect on different T-cell subsets. Vaccine. 1998;16:403–9. 9607063 10.1016/s0264-410x(97)80918-8

[25] Pinto LA, Edwards J, Castle PE, et al. Cellular immune responses to human papillomavirus (HPV)-16 L1 in healthy volunteers immunized with recombinant HPV-16 L1 virus-like particles. J Infect Dis. 2003;188:327–38. 12854090 10.1086/376505

[26] Emeny RT, Wheeler CM, Jansen KU, et al. Priming of human papillomavirus type 11-specific humoral and cellular immune responses in college-aged women with a virus-like particle vaccine. J Virol. 2002;76:7832–42. 12097595 10.1128/JVI.76.15.7832-7842.2002 PMC136358

[27] Lin C, Franceschi S, Clifford GM. Human papillomavirus types from infection to cancer in the anus, according to sex and HIV status: a systematic review and meta-analysis. Lancet Infect Dis. 2018;18:198–206. 29158102 10.1016/S1473-3099(17)30653-9 PMC5805865

[28] Riou C, du Bruyn E, Stek C, et al. Relationship of SARS-CoV-2-specific CD4 response to COVID-19 severity and impact of HIV-1 and tuberculosis coinfection. J Clin Invest. 2021;131:149125. 33945513 10.1172/JCI149125 PMC8203446

[29] Tortellini E, Zingaropoli MA, Mancarella G, et al. Quality of T-cell response to SARS-CoV-2 mRNA vaccine in ART-treated PLWH. Int J Mol Sci. 2022;23:14988. 36499317 10.3390/ijms232314988 PMC9741180

[30] Mullender C, da Costa KAS, Alrubayyi A, et al. SARS-CoV-2 immunity and vaccine strategies in people with HIV. Oxf Open Immunol. 2022;3:iqac005. 36846557 10.1093/oxfimm/iqac005 PMC9452103

[31] Overton ET, Sungkanuparph S, Powderly WG, et al. Undetectable plasma HIV RNA load predicts success after hepatitis B vaccination in HIV-infected persons. Clin Infect Dis. 2005;41:1045–8. 16142673 10.1086/433180

[32] Vergori A, Cozzi Lepri A, Cicalini S, et al. Immunogenicity to COVID-19 mRNA vaccine third dose in people living with HIV. Nat Commun. 2022;13:4922. 35995780 10.1038/s41467-022-32263-7 PMC9395398

[33] Woldemeskel BA, Karaba AH, Garliss CC, et al. The BNT162b2 mRNA Vaccine Elicits Robust Humoral and Cellular Immune Responses in People Living With Human Immunodeficiency Virus (HIV). Clin Infect Dis. 2021;74:1268–70. 10.1093/cid/ciab648 PMC8406881 34293114

[34] Agrati C, Gioia C, Castilletti C, et al. Cellular and humoral immune responses to pandemic influenza vaccine in healthy and in highly active antiretroviral therapy-treated HIV patients. AIDS Res Hum Retroviruses. 2012;28:1606–16. 22439734 10.1089/aid.2011.0371 PMC3505053

[35] Antinori A, Cicalini S, Meschi S, et al. Humoral and Cellular Immune Response Elicited by mRNA Vaccination Against Severe Acute Respiratory Syndrome Coronavirus 2 (SARS-CoV-2) in People Living With Human Immunodeficiency Virus Receiving Antiretroviral Therapy Based on Current CD4 T-Lymphocyte Count. Clin Infect Dis. 2022;75:e552–63. 10.1093/cid/ciac238 PMC9047161 35366316

[36] Kroon FP, van Dissel JT, de Jong JC, et al. Antibody response to influenza, tetanus and pneumococcal vaccines in HIV-seropositive individuals in relation to the number of CD4+ lymphocytes. AIDS. 1994;8:469–76. 7912086 10.1097/00002030-199404000-00008

[37] Freitas AA, Rocha BB. Lymphocyte lifespans: homeostasis, selection and competition. Immunol Today. 1993;14:25–9. 8442858 10.1016/0167-5699(93)90320-K

[38] Giannini SL, Hanon E, Moris P, et al. Enhanced humoral and memory B cellular immunity using HPV16/18 L1 VLP vaccine formulated with the MPL/aluminium salt combination (AS04) compared to aluminium salt only. Vaccine. 2006;24:5937–49. 16828940 10.1016/j.vaccine.2006.06.005

[39] Olsson S-E, Villa LL, Costa RLR, et al. Induction of immune memory following administration of a prophylactic quadrivalent human papillomavirus (HPV) types 6/11/16/18 L1 virus-like particle (VLP) vaccine. Vaccine. 2007;25:4931–9. 17499406 10.1016/j.vaccine.2007.03.049

[40] Roteli-Martins CM, Naud P, De Borba P, et al. Sustained immunogenicity and efficacy of the HPV-16/18 AS04-adjuvanted vaccine: up to 8.4 years of follow-up. Hum Vaccin Immunother. 2012;8:390–7. 22327492 10.4161/hv.18865

[41] Buggert M, Price DA, Mackay LK, et al. Human circulating and tissue-resident memory CD8+ T cells. Nat Immunol. 2023;24:1076–86. 37349380 10.1038/s41590-023-01538-6

[42] Lange J, Rivera-Ballesteros O, Buggert M. Human mucosal tissue-resident memory T cells in health and disease. Mucosal Immunol. 2022;15:389–97. 34743182 10.1038/s41385-021-00467-7 PMC8571012

