# Supplementary material for: Role of prior HPV infection and CD4 T-cell count in modulating cellular immune responses to a three-dose nonavalent HPV vaccine schedule in PWH receiving ART
Source: BMC Med. 2025 Dec 24;23:689. doi: 10.1186/s12916-025-04504-1 (PMC12729619; doi:10.1186/s12916-025-04504-1)
Supplement: Supplementary file 1 — Additional file 1: Table 1. [Demographic and clinical characteristics at baseline according to prior HPV infection and CD4 T-cell count at first HPV vaccine dose]. [file 12916_2025_4504_MOESM1_ESM.docx]

**Additional File 1: Table 1. Demographic and clinical characteristics at baseline according to (A) known previous HPV infection (HPV- vs HPV+) and (B) CD4 T-cell count at first HPV vaccine dose (≤500 CD4/µL vs >500 CD4/µL)**

| **(A)** | **HPV-** | **HPV+** | **p-value** |  |
| --- | --- | --- | --- | --- |
|  | **N=25 (65.8%)** | **N=13 (34.2%)** |  |  |
| **Sex, n (%)** |  |  |  |  |
| M | 19 (76.0%) | 10 (76.9%) | 0.949 |  |
| F | 6 (24.0%) | 3 (23.1%) |  |  |
| **Age, median [IQR]** | 42.0 [35.0 51.0] | 38.0 [34.0 46.0] | 0.612 |  |
| **Years from HIV diagnosis, median [IQR]** | 7.0 [3.0 10.0] | 11.0 [8.0 13.0] | 0.211 |  |
| **Calendar year 1^st^ vaccine dose, n (%)** |  |  |  |  |
| 2022 | 9 (36.0%) | 6 (46.2%) | 0.544 |  |
| 2023 | 16 (64.0%) | 7 (53.8%) |  |  |
| **CD4 count nadir, cells/µL, median [IQR]** | 309.0 [106.0 440.0] | 374.5 [89.0 577.0] | 0.528 |  |
| <200 cells/µL, n(%) | 7 (31.8%) | 4 (33.3%) | 0.928 |  |
| **Baseline CD4 count, cells/µL, median [IQR]** | 621.0 (442.0 845.0) | 850.0 [540.0 1188.0] | 0.162 |  |
| ≤500 CD4/µL, n (%) | 6 (24.0%) | 3 (23.1%) | 0.690 |  |
| >500 CD4/µL, n (%) | 19 (76.0%) | 10 (76.9%) |  |  |
| **Baseline HIV-RNA, cps/ml** |  |  |  |  |
| <50 cps/ml, n (%) | 22 (88.0 %) | 13 (100.0%) | 0.119 |  |
| ≥50 cps/ml, n (%) | 3 (12.0 %) | 0 (0.0%) |  |  |
| **CMV-IgG, n(%)** |  |  |  |  |
| Negative | 3 (12.0%) | 0 (0.0%) | 0.193 |  |
| Positive | 22 (88.0%) | 13 (100.0%) |  |  |
| **STI previous 6months, n (%)** |  |  |  |  |
| No | 24 (100.0%) | 10 (83.3%) | 0.040 |  |
| Yes | 0 (0.0%) | 2 (16.7%) |  |  |
| **Smoking, n(%)** |  |  |  |  |
| Unknown | 3 (12.0%) | 0 (0.0%) | 0.404 |  |
| No | 8 (32.0%) | 4 (30.8%) |  |  |
| Yes | 14 (56.0%) | 9 (69.2%) |  |  |
| **Comorbidities, n(%)** |  |  |  |  |
| No | 18 (72.0%) | 10 (76.9%) | 0.744 |  |
| Yes | 7 (28.0%) | 3 (23.1%) |  |  |
| **ART class, n(%)** |  |  |  |  |
| 2DR | 10 (40.0%) | 4 (30.8%) | 0.617 |  |
| 3DR | 14 (56.0%) | 9 (69.2%) |  |  |
| Other | 1 (4.0%) | 0 (0.0%) |  |  |
|  |  |  |  |  |
| **(B)** | **CD4 ≤500 cells/µL** | **CD4 >500 cells/µL** | **p-value** |  |
|  | **N=9 (24.0%)** | **N=29 (76.0%)** |  |  |
| **Sex, n (%)** |  |  |  |  |
| M | 8 (88.9%) | 21 (72.4%) | 0.216 |  |
| F | 1 (11.1%) | 8 (27.6%) |  |  |
| **Age, median [IQR]** | 37.0 [34.0 42.0] | 42.0 [34.0 52.0] | 0.393 |  |
| **Years from HIV diagnosis, median [IQR]** | 6.0 [3.0 8.0] | 10.0 [4.0 12.0] | 0.281 |  |
| **Calendar year 1^st^ vaccine dose, n (%)** |  |  |  |  |
| 2022 | 3 (33.3%) | 12 (41.4%) | 0.967 |  |
| 2023 | 6 (66.7%) | 17 (58.6%) |  |  |
| **Previous HPV infection, n (%)** |  |  | 0.690 |  |
| No | 6 (66.7%) | 19 (65.5%) |  |  |
| Yes | 3 (33.3%) | 10 (34.5%) |  |  |
| **CD4 count nadir, cells/µL, median [IQR]** | 73.0 [26.0 165.0] | 392.0 [278.0 589.0] | 0.019 |  |
| <200 cells/µL, n(%) | 7 (77.8%) | 4 (16.0%) | <0.001 |  |
| **Baseline CD4 count, cells/µL, median [IQR]** | 369.0 (269.0 419.0) | 826.0 [605.0 1126.0] | <0.001 |  |
| **Baseline HIV-RNA, cps/ml** |  |  |  |  |
| <50 cps/ml, n (%) | 8 (88.8%) | 27 (93.1%) | 0.273 |  |
| ≥50 cps/ml, n (%) | 1 (11.1%) | 2 (6.9%) |  |  |
| **CMV IgG, n (%)** |  |  |  |  |
| Negative | 1 (11.1%) | 2 (6.9%) | 0.798 |  |
| Positive | 8 (88.9%) | 27 (93.1%) |  |  |
| **STI previous 6months, n (%)** |  |  |  |  |
| No | 9 (100.0%) | 25 (93.1%) | 0.367 |  |
| Yes | 0 (0.0%) | 2 (6.9%) |  |  |
| **Smoking, n (%)** |  |  |  |  |
| Unknown | 1 (11.1%) | 2 (6.9%) | 0.616 |  |
| No | 2 (22.2%) | 10 (34.5%) |  |  |
| Yes | 6 (66.7%) | 17 (58.6%) |  |  |
| **Comorbidities, n (%)** |  |  |  |  |
| No | 6 (66.7%) | 22 (75.9%) | 0.804 |  |
| Yes | 3 (33.3%) | 7 (24.1%) |  |  |
| **ART class, n (%)** |  |  |  |  |
| 2DR | 1 (11.1%) | 13 (44.8%) | 0.128 |  |
| 3DR | 7 (77.8%) | 16 (55.2%) |  |  |
| Other | 1 (11.1%) | 0 (0.0%) |  |  |
| *Abbreviations: M, male; F, Female; HPV, human papillomavirus;CMV, cytomegalovirus; STI, sexually transmitted infections; ART, antiretroviral therapy; 2DR, two-drug regimen; 3DR, three-drug-regimen* | | | | |
