# Supplementary material for: Role of prior HPV infection and CD4 T-cell count in modulating cellular immune responses to a three-dose nonavalent HPV vaccine schedule in PWH receiving ART
Source: BMC Med. 2025 Dec 24;23:689. doi: 10.1186/s12916-025-04504-1 (PMC12729619; doi:10.1186/s12916-025-04504-1)
Supplement: Supplementary file 3 — Additional file 3: Table 1. [Post-hoc power analysis on changes on immune response according to CD4 strata]. [file 12916_2025_4504_MOESM3_ESM.docx]

**Additional File 3: Table 1**. Post-hoc power analysis on changes on immune response according to CD4 strata.

| Marker | Timepoint | Mean change group CD4≤500/µL | Mean change group CD4>500/µL | Between-groups difference (delta) | Power |
| --- | --- | --- | --- | --- | --- |
| CD4-responding | T1–T0 | –0.176 | +1.113 | 1.289 | 0.37 |
|  | T2–T0 | +0.929 | +2.004 | 1.075 | 0.28 |
| CD4-polyfunctional | T1–T0 | 0.000 | +0.058 | 0.058 | 0.32 |
|  | T2–T0 | +0.011 | +0.168 | 0.157 | 0.98 |
| CD8-responding | T1–T0 | +0.036 | +1.612 | 1.576 | 0.59 |
|  | T2–T0 | +0.290 | +2.694 | 2.404 | 0.91 |
| CD8-polyfunctional | T1–T0 | 0.000 | +0.042 | 0.042 | 0.20 |
|  | T2–T0 | +0.009 | +0.179 | 0.170 | 0.99 |

Mean changes represent the observed unadjusted mean changes within group from T0. Power was estimated using *power twomeans* in Stata with an α=.05, assuming equal standard deviation (SD) between groups (based on pooled SD) with a sample size of n=9 for the CD4≤500/µL group and n=29 for the CD4>500/µL group.
